# Empowering patients with comorbid diabetes and hypertension through a multi-component intervention of mobile app, health coaching and shared decision-making: Protocol for an effectiveness-implementation of randomised controlled trial

**DOI:** 10.1371/journal.pone.0296338

**Published:** 2024-02-26

**Authors:** Yu Heng Kwan, Sungwon Yoon, Bee Choo Tai, Chuen Seng Tan, Jie Kie Phang, Wee Boon Tan, Ngiap Chuan Tan, Cynthia Yan Ling Tan, David Koot, Yan Ling Quah, Hock Hai Teo, Lian Leng Low

**Affiliations:** 1 Centre for Population Health Research and Implementation (CPHRI), SingHealth Regional Health System, SingHealth, Singapore, Singapore; 2 Health Services and Systems Research, Duke-NUS Medical School, Singapore, Singapore; 3 SingHealth Internal Medicine Residency Programme, Singapore, Singapore; 4 Saw Swee Hock School of Public Health, National University of Singapore and National University Health System, Singapore, Singapore; 5 Yong Loo Lin School of Medicine, National University of Singapore and National University Health System, Singapore, Singapore; 6 SingHealth Polyclinics, Singapore, Singapore; 7 SingHealth Duke-NUS Family Medicine Academic Clinical Program, Duke-NUS Medical School, Singapore, Singapore; 8 School of Computing, National University of Singapore, Singapore, Singapore; 9 Population Health & Integrated Care Office (PHICO), Singapore General Hospital, Singapore, Singapore; 10 SingHealth Community Hospital, Singapore, Singapore; 11 Department of Family Medicine & Continuing Care, Singapore General Hospital, Singapore, Singapore; National Healthcare Group, SINGAPORE

## Abstract

**Introduction:**

Diabetes and hypertension are prevalent and costly to the health system. We have developed a mobile app (EMPOWER app) which enables remote monitoring and education through personalised nudges. We aim to study the effectiveness of a multi-component intervention comprising the EMPOWER mobile app with health coaching and shared decision-making for diabetes and hypertension.

**Methods:**

We will conduct a two-arm, open-label, pragmatic randomised controlled trial (RCT). Participants with comorbid diabetes and hypertension enrolled from public primary care clinics will be randomised to either intervention or control in a 1:1 ratio. The intervention group participants will have access to health coaching with shared decision-making interventions in addition to the EMPOWER app and their usual primary care. The control group participants will continue to receive usual primary care and will neither receive the EMPOWER app nor health coaching and shared decision-making interventions. Our primary outcome is change in HbA1c level over 9 months. Secondary outcomes include change in systolic blood pressure, quality of life, patient activation, medication adherence, physical activity level, diet, and healthcare cost (direct and indirect) over 9 months.

**Discussion:**

Our trial will provide key insights into clinical- and cost-effectiveness of a multi-component intervention comprising EMPOWER mobile app, health coaching and shared decision-making in diabetes and hypertension management. This trial will also offer evidence on cost-effective and sustainable methods for promoting behavioural changes among patients with comorbid diabetes and hypertension.

**Trial registration:**

This study was registered on clintrials.gov on August 3, 2022, with the trial registration number: NCT05486390.

## Introduction

Diabetes and hypertension are prevalent chronic diseases worldwide, with 537 million adults having diabetes worldwide [1] and the age-standardised prevalence of hypertension being around 30% globally [2]. Moreover, these diabetic and hypertensive patients are at greater risk for microvascular and macrovascular complications [3], including elevated rates of cardiovascular death and myocardial infarction [4]. It is well-established that lifestyle improvements in dietary habits and physical activity are effective in improving outcomes of both diabetes [5] and hypertension [6, 7]. However, due to the time constraints [8, 9], promotion of healthier lifestyle changes in conventional healthcare settings is difficult [10].

Mobile health (mHealth) can be an effective solution to promote lifestyle modification among patients with comorbid diabetes and hypertension. Previous reviews have shown that mHealth interventions may be effective for improving patient outcomes among patients with diabetes or hypertension [11, 12]. However, limited evidence exists for effectiveness of mHealth intervention for patients with comorbid diabetes and hypertension [11, 13]. We have developed a novel mobile app (EMPOWER app) which enables remote monitoring and education of patients with diabetes through personalised nudges. The effectiveness and implementation of the EMPOWER app in improving diabetes self-management is being evaluated in an ongoing randomised controlled trial [14]. However, numerous trials have shown that patient self-monitoring alone may have a limited effect on improving blood pressure control for patients with comorbid conditions [15–17]. To effectively combat the comorbid condition of inadequate blood pressure management [18, 19], it is important to develop an innovative care model that involves multiple components including mHealth solution, health coaching and shared decision making.

Therefore, we aim to study the effectiveness of the EMPOWER mobile app in conjunction with health coaching and shared decision-making in patients with comorbid diabetes and hypertension through a randomised controlled trial (RCT). The findings from this study may provide evidence for better management and care model for patients with comorbid diabetes and hypertension. This protocol paper details the study design and planned analyses, and is based on protocol version 2 dated 1 October 2022.

## Methods

### Study design

**Fig 1:**
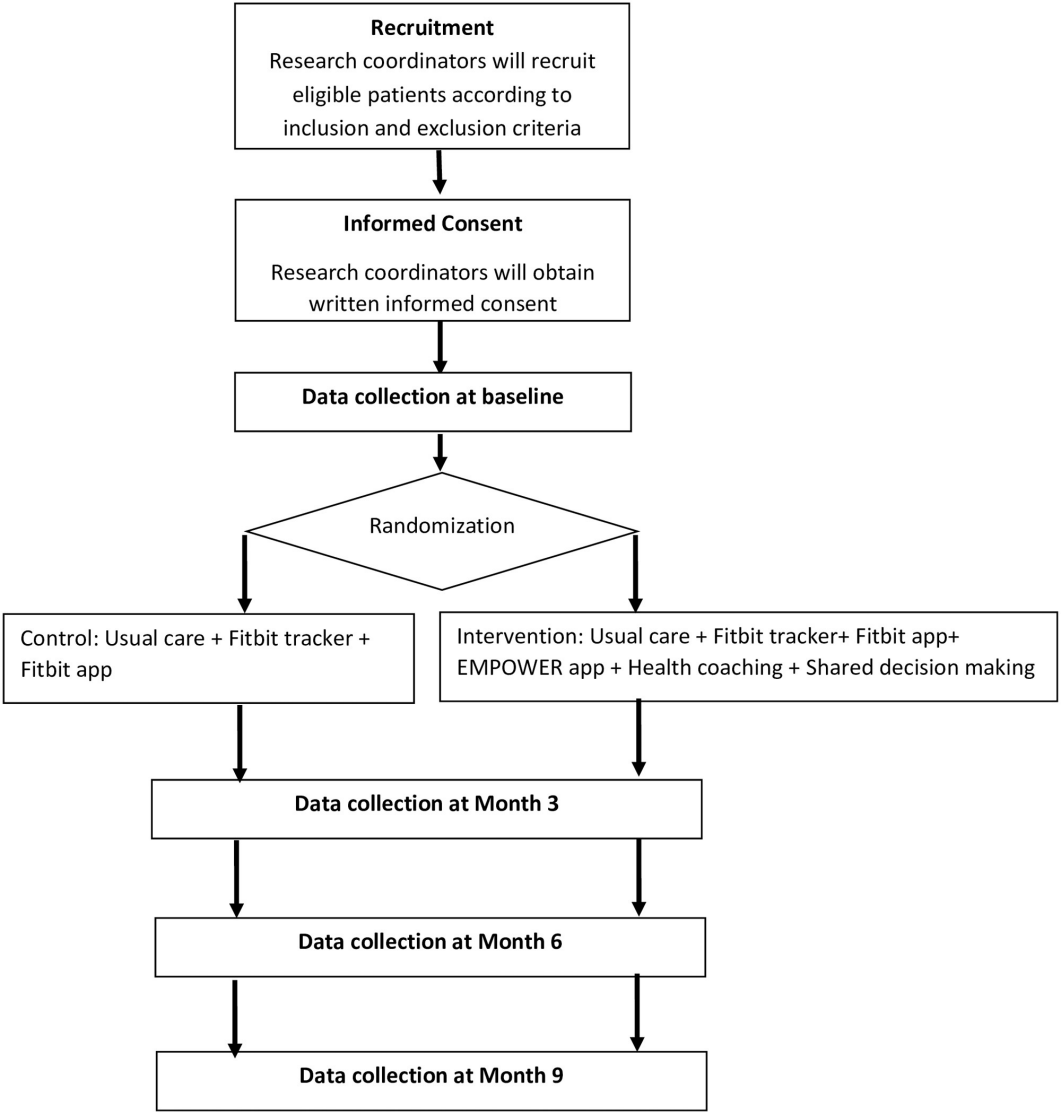
Trial work plan. Follow- up will be performed at month 3, 6 and 9.

**Fig 2:**
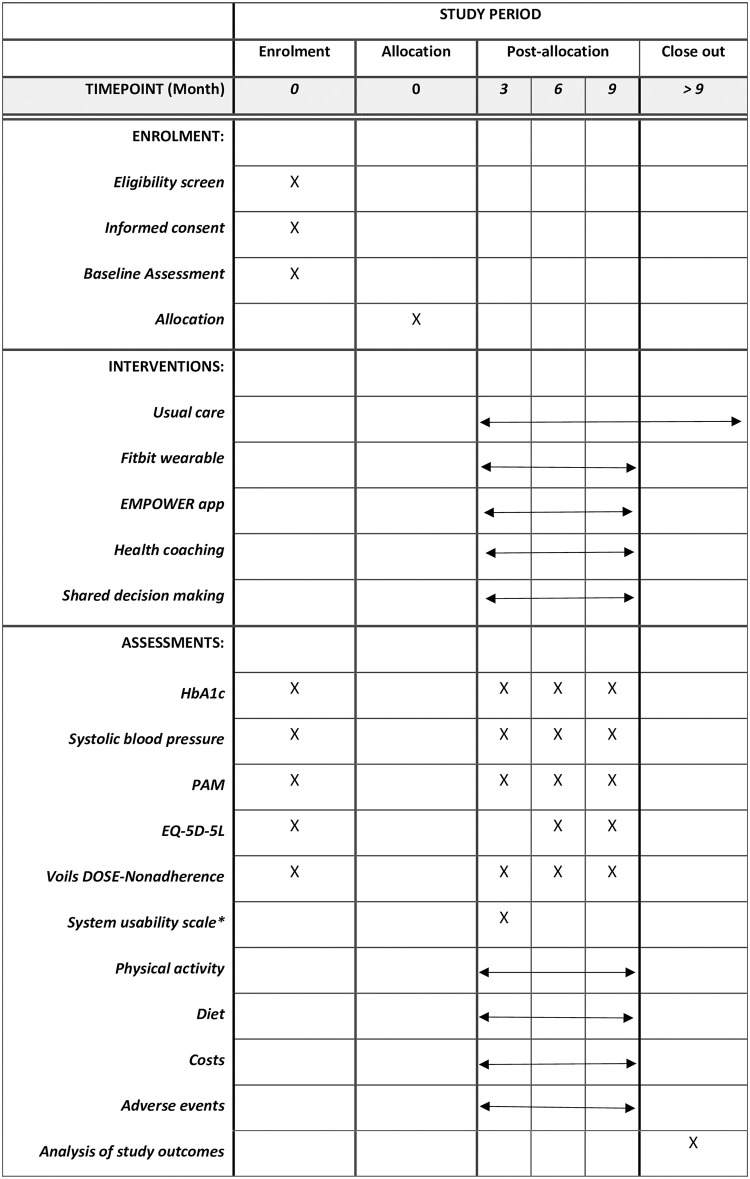
SPIRIT figure for the schedule of enrolment, interventions, and assessments. Abbreviation: EQ-5D-5L: EuroQoL-5 Dimensions; HbA1c: Haemoglobin A1c; PAM: Patient Activation Measure. * Only for participants randomized to intervention group.

We will execute an open label, two-arm, parallel randomised pragmatic trial. The pragmatic study design is based on the Pragmatic Explanatory Continuum Indicator Summary Framework-2 (PRECIS-2) criteria [20]. We will randomly allocate eligible and consented participants in a 1:1 ratio to either intervention or control group. The intervention participants will have access to (1) EMPOWER mobile app, (2) Fitbit tracker and Fitbit app, (3) health coaching, (4) shared decision making, and (5) usual primary care. The control group participants will get Fitbit tracker, Fitbit app and usual primary care only (Fig 1). This study protocol is written according to the Standard Protocol Items: Recommendations for Interventional Trials (SPIRIT) reporting guidelines [21] (Fig 2 and S1 File: SPIRIT 2013 Checklist). Participant recruitment has commenced on 17 February 2023, but has not been completed at the time of manuscript submission and publication.

### Ethics and dissemination

Ethics approval was granted (Reference number: 2022/2326) by the SingHealth Centralised Institutional Review Board. In addition, the board oversees protocol amendment, adverse event reporting and review. Written informed consent will be obtained from all participants prior to study commencement. The informed consent document can be found in the S2 File: Consent form. Payment and compensation of injuries and illness to participants arising from participation in this study will be assessed by the study team, and will be covered as per guidelines under the National Clinical Trials Insurance policy that SingHealth subscribes to.

Study findings will be disseminated through presenting at international conferences as well as publishing in peer-reviewed journals. The authorship will be finalised by the overall principal investigator (Lian Leng Low) according to the International Committee of Medical Journal Editors (ICMJE) guidelines. The study team does not intend to engage professional writers for this study.

### Inclusion and exclusion criteria

Patients are recruited from three polyclinics in Singapore, which are public healthcare centres providing subsidised primary care. The trial inclusion criteria at the time of recruitment are:

(1) Aged 21 years and older,
(2) Has a diagnosis of diabetes,
(3) Haemoglobin A1c (HbA1c) ≥ 7.0% (most recent result),
(4) Systolic blood pressure≥ 140 mmHg or diastolic blood pressure ≥ 90 mmHg on two or more prior visits, physician-diagnosed hypertension, or on antihypertensive medication,
(5) Able to exercise physically,
(6) Literate in English.

We will exclude patients who at the time of recruitment:

(1) Require aid with basic activities of daily living,
(2) On bolus insulin,
(3) Have upcoming major medical or surgical procedure during the trial,
(4) Impaired cognitively [22].

### Intervention

The EMPOWER app has been described in detail in an earlier paper (14). The key features and functionalities of the EMPOWER app include (1) Personalised nudges based on Artificial Intelligence (AI) learning of the participant’s behaviour, (2) Gamification, (3) Logging and report, (4) Educational resources, which are mapped to key domains and interventions in the Behavioural Change Wheel (BCW) (S3 File: EMPOWER app features) [23].

The intervention group participants will interact with health coach throughout the study duration. The health coach involved in this study will include a registered nurse under the telecare service unit in an acute hospital. The health coach will contact the intervention group participants via phone call, videocall and/ or text messages. In this study, the health coach will be responsible for the following roles [24]:

(1) Goal setting and action plan: Health coach will discuss the goals of treatment, and the reason for these goals. The health coach will discuss lifestyle factors influencing response to treatment of diabetes, hypertension and/or hyperlipidemia (e.g., salt or carbohydrate intake, weight, and physical activity). The health coach will also discuss with the participants regarding their willingness to modify these lifestyle factors, and propose a customised action plan for each participant. The health coach will also prepare a tailored education program consisting of information about the risks of diabetes, hypertension and/or hyperlipidemia as well as the advantages of treatment and lifestyle changes.
(2) After-visit summary: After consultation with physician, health coach will ask participants whether there is anything they would like to discuss. Health coach will generate an after-visit summary to recap the advice given by the physician during the visit, including behaviour changes plan, medication plan, and subsequent follow-up date. Health coach will also make sure that participants are able to navigate the health system to accomplish the agendas in the after-visit summary.

A report card summarising the lifestyle behaviour (including diet, physical activity and weight), goals and action plans will be made available in the app to facilitate shared decision making.

### Control

The control group participants will get Fitbit tracker and the Fitbit app but will have no access to the EMPOWER app, health coach nor shared decision making. This will allow investigation of the effectiveness of the EMPOWER mobile app in conjunction with health coaching and shared decision making in patients with comorbid diabetes and hypertension.

### Outcome measures

Our primary outcome is change in HbA1c level over 9 months (Table 2). The measurement of glycated haemoglobin using HbA1c is widely accepted as a standard index of glycaemic control of diabetic patients [25]. HbA1c will be measured using platforms available at the in-house laboratory at the study sites, and the platforms will be National Glycohemoglobin Standardization Program-certified.

Our secondary outcomes will include change in systolic blood pressure, quality of life (QoL), patient activation, medication adherence, physical activity level, diet, and healthcare cost (direct and indirect) over 9 months. The outcome measures are listed in Table 1. We will extract healthcare cost from electronic medical records, and supplement with survey data on self-reported income, travel cost, and employment status.

**Table 1:** Outcome measures to evaluate clinical and cost-effectiveness.

Variables	Source and Measure/ Instrument name	Time of collection
Primary Outcome Measure		
Haemoglobin A1c (HbA1c)	Platforms available at the in-house laboratory at polyclinics	Baseline, 3, 6, and 9 months
Secondary Outcome Measures		
Systolic blood pressure	Devices available at polyclinics	Baseline, 3, 6, and 9 months
Physical activity	Smartwatch: steps taken, active minutes, sleep quality, sleep time and heart rate	Baseline (first 2 weeks), and real-time tracking throughout 9-month follow-up
Dietary intake	Mobile application: Calorie intake, carbohydrate intake and sodium intake	Baseline (first 2 weeks), and real-time tracking throughout 9-month follow-up
Patient activation	Survey: Patient activation measure (PAM) [26]	Baseline, 3, 6, and 9 months
Medication adherence	Survey: Voils DOSE-Nonadherence [27]	Baseline, 3, 6, and 9 months
Quality of life	Survey: EQ-5D-5L [28]	Baseline, 6, and 9 months
Direct healthcare cost	Electronic medical records: Costs of consultations, lab tests, medications, admissions	All entries throughout 9-month follow-up
Indirect healthcare cost	Survey: Self-reported income, travel cost, employment status	Baseline, 3, 6, and 9 months

### Sample size and recruitment

We calculated the sample size based on our primary outcome of HbA1c which is evaluated at baseline, 3 months, 6 months and 9 months. We assume a mean difference of 0.36%, a standard deviation of 1.5% [29], and an auto-correlation of 0.375 [30] between the repeated HbA1c measurements. The standard deviation of 1.5% was derived from data from the SingHealth Diabetes Registry, which showed the standard deviation of HbA1c for patients from polyclinics, hospitals and national specialty centres ranging from 1.4–1.5 from 2013–2019 [29]. Conservatively, we postulated that effect size of a complex intervention involving health coach and mobile app would result in a mean difference of 0.36% in HbA1c levels between the intervention and control groups. A meta-analysis has shown that the mean reduction in HbA1c is 0.41% for high quality studies involving mobile applications for Type 2 diabetes [31]. The auto-correlation of 0.375 between the repeated HbA1c measurements was assumed as it provided the most conservative (i.e. maximum) estimate of the sample size based on a repeated measurement study design with one-pre and three-post intervention measurements [30].

Based on a repeated measures study design [30] with one pre- and three post-intervention measurements, a minimum sample size of 125 per group, or 250 in total would have an 80% power to detect the hypothesised difference at a two-sided alpha of 5%. A total of 320 participants will be required after accounting for 20% attrition.

We will be advertising the study through posters and banners placed at polyclinic sites to ensure adequate enrolment to reach the target sample size of 320.

### Randomization and blinding process

Participants will be randomised to intervention or control group using a computer-generated randomization list provided by the trial biostatistician. We implemented stratified block randomization using randomly varying block sizes with recruitment site and duration of diabetes (<10 years or ≥10 years) as stratification factors based on a 1:1 allocation ratio. The cut-off of 10 years for duration of diabetes is supported by the data from previous studies [32, 33] and local SingHealth Diabetes Registry (mean disease duration ranging from 9.0–11.3 years from 2013 to 2019) [34].

Upon obtaining informed consent and enrolling a participant, the research coordinators at the polyclinic sites will contact a centralised research staff (who oversees the study progress but is not involved in patient recruitment) for the randomisation assignment.

The data analyst will be blinded to the assignment of interventions. It is not possible to blind study participants and care providers to the random assignment due to the nature of the intervention in this study.

### Statistical analyses

We will summarise participant’s characteristics using median (interquartile range) for non-normally distributed or mean (standard deviation) for normally distributed continuous variables respectively, and count and percentage for categorical variables. We will analyse the primary outcome of change in HbA1c over the 9 months follow-up, using a linear mixed model with adjustment for baseline HbA1c. A further model will be considered to explore whether the treatment effect on HbA1c varies with time, by including the treatment by time interaction, and adjusting for baseline systolic blood pressure and other potential confounders as appropriate via the mixed model.

We will use linear mixed model to account for within-individual correlation among measurements, and the sandwich estimator to obtain robust standard error estimates for secondary outcomes with repeated measurements (e.g., systolic blood pressure, quality of life, patient activation score). Pseudo maximum likelihood estimation method will be used for the parameter estimation. We will include intervention indicator and time factor as predictors, adjusting for the respective baseline covariates. For both the primary and secondary outcomes, alternative models such as mixed effect quantile regression may be considered if the model assumptions are violated.

All evaluations will be made using two-sided p-values at the 5% level of significance. Statistical analyses will be conducted based on the principle of intention-to-treat.

We will also conduct an economic evaluation from the health care system and societal perspectives. We will perform both cost-effectiveness analysis (i.e., cost of reduction in 1% in HbA1c) and cost-utility analysis (cost of reduction in 1 quality-adjusted life year saved).

### Implementation

We will assess the impact and sustainability of interventions using the RE-AIM framework [35]. Data sources include qualitative focus groups and interviews with a purposive sample of participants and stakeholders, survey data, and electronic medical records. In addition, qualitative interviews will be conducted with a purposive sample of participants to collect their feedback on the EMPOWER app, health coaching and shared decision making interventions in promoting behavior change based on shared domains of the theoretical domain framework [36, 37] and social cognitive theory [38]. Specifically, we will focus on exploring knowledge, skills and reinforcement, self-efficacy, beliefs about consequences (outcome expectation), intention and goals, environmental context and social support/influences (S4 File: Sample interview questions for patients). These domains will assist in identifying cognitive, affective and social/environmental factors influencing behavioral change. In addition to patient interviews, interviews with healthcare professionals (clinician, health coach) and research coordinators will be conducted to elucidate implementation challenges and experiences (S5 File: Sample interview questions for other stakeholders). The details on data sources and parameters assessed for each RE-AIM dimension can be found in Table 2.

**Table 2:** Data sources and parameters assessed for RE-AIM dimensions.

RE-AIM Dimension	Data source	Parameters
Reach	Demographic data, administrative data, RCT survey data, interviews with stakeholders	Estimated number and percentage of patients with comorbid diabetes and hypertension approached Estimated number and percentage of patients eligible for the study Reasons for refusal to participate Number and percentage of dropouts, and reasons
Effectiveness	RCT survey data, EMR	Clinical measures before and after the intervention: HbA1c to target BP readings to target Long term complications (e.g., Major Adverse Cardiac Events, death, surgery, stroke, amputations, renal failure) Patient activation (Patient activation measure [26]) Physical activity (Wearable data) Medical Adherence (Voils DOSE-Nonadherence [27] and medication logging in EMPOWER app) Dietary changes (Diet logging in EMPOWER app) Quality of life (EQ-5D [28]) Length of stay, hospital-free days Direct healthcare cost, indirect healthcare cost
Adoption	Interviews with stakeholders	Interest of institutions and clinicians to adopt the interventions Proportion of patients expressed interest in continuing interaction with health coach Support from relevant partners and staff members
Implementation	Fidelity checks, RCT survey data, interviews with patients and stakeholders	Patient compliance to monitoring schedule Patient feedback on intervention (EMPOWER app, health coaching and shared decision making) Factors facilitate or impede implementation Adherence to protocol by health coach
Maintenance	Administrative data, Interviews with patients and stakeholders, survey data	Usage of EMPOWER app after completion of RCT Usage of smartwatch after completion of RCT Clinical outcomes at 3 and 6 months after completion of RCT Patients feedback on sustained usage of EMPOWER app and smartwatch

Abbreviation

BP: Blood pressure; EMR: Electronic medical records; HbA1c: Haemoglobin A1c; RCT: Randomised controlled trial

### Data management

We will secure the hardcopy survey data in locked storage spaces at the respective recruitment sites. Research coordinators will enter data into a secure web application [39, 40]. We will secure the EMPOWER app data and Fitbit data on a password-protected cloud, where only study team members will have access. We will retrieve data from the electronic medical records upon participant’s consent. The principal investigator will oversee data entry, data monitoring and audit procedures. Only the principal investigator and site principal investigators approved by the institutional review board will have access to the final trial dataset. The authors have access to information that could identify individual participants during and after data collection.

### Data monitoring

A data and safety monitoring board (DSMB) will ensure proper conduct of this trial and patient safety. At 6-month of completed follow up for 160 participants, the independent DSMB will conduct a planned monitoring of the unblinded effectiveness and safety data. All adverse events will be recorded.

## Discussion

This study will be the first to report on the effectiveness and implementation of complex intervention including personalised nudges driven by AI, health coaching and shared decision-making informed by behaviour change theory [41] for comorbid diabetes and hypertension management. Our study is conducted in an Asian healthcare setting, which will add much needed evidence to prior mHealth studies on chronic disease self-management that have been conducted primarily in the context of Western healthcare settings [42, 43]. Previous studies have demonstrated the importance of cultural adaptations of healthcare interventions to their effectiveness [44, 45]. The implementation of digitalisation and automation of nudging via mobile application, health coaching and shared decision-making in this study has the potential to alleviate time and resource constraints in primary care [9, 46, 47].

Our hybrid implementation-effectiveness study will provide valuable insights into a novel care model comprising mHealth and health coaching in the management of chronic diseases such as diabetes and hypertension. The evaluation of interventions using the RE-AIM framework [35] as well as feedback on the multi-component intervention (comprising EMPOWER app, health coaching and shared decision making) in promoting behavior change based on shared domains of theoretical domain framework [36, 37] and social cognitive theory [38] will generate findings on both impact and sustainability. Clinicians, researchers and policy makers will glean insights as to how cost-effective mHealth, health coaching and shared decision-making interventions can be scaled up and sustained among patients with comorbid diabetes and hypertension for behavioural change.

## Supporting information

S1 File SPIRIT 2013 checklist. (DOCX)

S2 File Consent form. (DOCX)

S3 File EMPOWER app features. (DOCX)

S4 File Sample interview questions for patients. (DOCX)

S5 File Sample interview questions for other stakeholders. (DOCX)
