# Supplementary material for: Empowering patients with comorbid diabetes and hypertension through a multi-component intervention of mobile app, health coaching and shared decision-making: Protocol for an effectiveness-implementation of randomised controlled trial
Source: PLoS One. 2024 Feb 26;19(2):e0296338. doi: 10.1371/journal.pone.0296338 (PMC10896544; doi:10.1371/journal.pone.0296338)
Supplement: S2 File — (DOCX) [file pone.0296338.s002.docx]

Supplementary information 2: Consent form

**PARTICIPANT INFORMATION SHEET AND CONSENT FORM**

You are being invited to participate in a research study. Your participation in this study is entirely voluntary. Before you take part in this research study, the study must be explained to you and you must be given the chance to ask questions. Your questions will be answered clearly and to your satisfaction. Please read carefully the information provided here. If you agree to participate, please sign the consent form. You will be given a copy of this document.

**STUDY INFORMATION**

**Protocol Title:**

EMPOWERing patients with chronic diseases through smartphone app, health coaching and shared decision-making

**Principal Investigator:**

A/Prof Low Lian Leng

Department of Family Medicine & Continuing Care

Singapore General Hospital

**Site-Principal Investigator (Bedok):**

Dr Tan Yan Ling Cynthia

SingHealth Polyclinic (Bedok)

**Site-Principal Investigator (Punggol):**

Dr Quah Yan Ling

SingHealth Polyclinic (Punggol)

**Site-Principal Investigator (Tampines):**

Dr David Koot

SingHealth Polyclinic (Tampines)

**PURPOSE OF THE RESEARCH STUDY**

There is a rising prevalence of diabetes and hypertension in Singapore. The use of wearable technology such as smartphone wearable tracker and mobile applications for chronic disease management can potentially improve diabetes and hypertension control. We are also introducing a health coach that would assist you in shared decision-making of treatment and management packages for your diabetes and hypertension based on clinical evidence and your informed preferences.

The purpose of this study is to understand the clinical effectiveness, safety, and cost-effectiveness of smartphone wearable tracker with EMPOWER mobile health application, health coaching, and shared decision making in the management of diabetes and hypertension. We hope to understand whether the use of EMPOWER mobile app with personalized reminders based on an individual’s lifestyle will result in better management of symptoms in patients with diabetes. We also hope to explore the shared decision-making model for targeted chronic disease care and the inclusion of a health coach in the process aims to improve patient empowerment and ownership towards individual health conditions.

You were selected as a possible participant in this study because you have been diagnosed with diabetes and hypertension.

This study targets to recruit 320 participants from SingHealth Polyclinics over a period of one year.

**STUDY PROCEDURES & YOUR RESPONSIBILITIES IN THIS STUDY**

If you agree to participate in this study, you should:

- Wear the smartphone wearable tracker as often as possible
- Keep to the schedule that has been given to you along with the smartphone wearable tracker during the handover.
- Inform the Clinical Research Coordinator (CRC) immediately in the event that the Smartphone wearable tracker is lost and/or damaged.
- Adhere to the management plans communicated with the health coach and be involved in the shared decision-making process.

If you agree to take part in this study, you will be randomised to either the Intervention Arm or Control Arm. Participants randomised to the Control Arm will receive a smartphone wearable tracker with built-in tracking technologies. Participants randomised to the Intervention Arm will receive a smartphone wearable tracker with the EMPOWER mobile app that gives personalised reminders based on your lifestyle installed, on top of the built-in tracking technologies. Additionally, they will be assigned a health coach and be part of a shared decision-making model as proposed by the study. Randomisation means assigning you to one of two groups by chance, like tossing a coin or rolling a dice. Only you and the CRC will know which group you have been assigned to. If it becomes necessary for your care, your study doctor will be notified whether you are in the Control Arm or Intervention Arm.

If you agree to take part in this study, we would like to seek your permission to use your clinical data (e.g. gender, height, weight, ethnicity, the severity of disease, medications, appointments, and lab results) for research purposes. We will also collect data of costs incurred by you in using services in SingHealth Polyclinics (e.g. consultation costs, costs of laboratory tests, radiological tests, and medication costs) from the business office in SingHealth Polyclinics. We would like to seek your permission to use your smartphone wearable tracker data (physical activity, sleep, heart rate). If you are randomised to the Intervention Arm, we would like to seek your permission to access your app usage activity, as well as data that has been logged into the EMPOWER mobile app (e.g. meal logs, medication logs).

If you agree to take part in this study, your participation will last for 18 months, and you will be asked to complete 4 questionnaires throughout the study. You will be required to complete a questionnaire on the day of enrolment, at the 3-month mark, at the 6-month mark, and at the 9-month mark of the study. Each questionnaire is estimated to take 30 minutes to complete and will be done during your routine clinical visit or the Clinical Research Coordinator (CRC) will contact you via phone. Questionnaire include questions on socio-demographics, medication adherence, quality of life, patient activation, and economic outcomes. There will only be passive data collection on clinical data (e.g., HbA1c, blood pressure, cholesterol) from our medical records and app usage beyond 9 months.

1. **Smartphone wearable tracker with EMPOWER mobile application**

You will be either given a smartphone wearable tracker with the EMPOWER mobile app installed along with the in-built technologies or given a Smartphone wearable tracker with the in-built technologies. For patients randomised to Intervention Arm, you will be asked to fill in a System Usability Survey either during your 3-month routine clinical visit, or the CRC will contact you via phone. You may be contacted by the CRC for updates regarding the usage of the device. If you are being randomised to Control Arm, there will be no change in your care at all.

1. **Health Coaching and Shared Decision-making**

For patients randomised to Intervention Arm, you will also be assigned a health coach and be part of a shared decision-making model in this study. For the current study, shared decision-making involves the patient, health coach and the physicians to jointly offer treatment, care and support packages based on clinical evidence and the patients’ informed preferences. The health coach may be a trained nurse or a clinical staff with knowledge in diabetes and hypertension. By participating in the current study, you have consented to the assignment of a health coach to you and be part of a shared-decision model for your diabetes and hypertension management. The health coach will be contacting you through phone, WhatsApp or videocall (Zoom) throughout the first 9-month period. Your contact number and medication plan will be shared with health coach to facilitate the health coaching process.

If you agree to participate in this study, you should follow the advice and directions given to you by the study team.

**WHAT IS NOT STANDARD CARE OR IS EXPERIMENTAL IN THIS STUDY**

The study is being conducted because the use of the EMPOWER mobile app with smartphone wearable tracker, health coaching and shared decision-making as means to manage diabetes and hypertension have not been widely explored in Singapore yet. We hope that your participation will help us to determine whether additional interventions in the form of mobile apps (in particular, personalized nudges), health coaching and shared decision-making are beneficial to the existing routine care.

Standard care refers to the routine clinical care that you receive in SingHealth Polyclinics concerning your diabetes management. The use of EMPOWER mobile app with smartphone wearable tracker is not part of standard care in SingHealth Polyclinics (i.e. no smartphone wearable tracker is involved in your routine clinical care). Additionally, the involvement of a health coach in the shared decision-making process is also not part of standard care in SingHealth Polyclinics.

**POSSIBLE RISKS, DISCOMFORTS OR INCONVENIENCES**

For both intervention and control groups, there should be no additional risk or discomfort as all data collection will be part of your routine clinical care.

No additional visits to SingHealth Polyclinics will be required for this study. However, the involvement of a health coach may result in inconveniences to your usual chronic disease care routine as the health coach will contact you through phone or video call to aid in your chronic disease management. We hope to obtain your understanding that participation in the current study cannot proceed without the involvement of the health coach and being included in the shared decision-making model. The health coach will arrange with you for a suitable time to call. Therefore, in the event that you wish to not continue with the programme, you may approach the Clinical Research Coordinator (CRC) for study withdrawal.

**Personal privacy and confidentiality:**

This study uses health information that may affect your privacy. To protect your confidentiality, only a unique code number will be used to identify data we collected from you.

As there will be a link between the code and your identifiable information, there is still a possibility of a data breach. A data breach is when someone sees or uses data without permission. If there is a data breach, someone could see or use the data we have about you. Even without your name, there is a chance someone could figure out who you are. They could misuse your data. We believe the chance of this is very small, but it is not zero.

**Questionnaires/surveys:**

Some of the questions might make you feel uncomfortable or upset. You may decline to answer any of the questions and/or take a break at any time during the study.

**POTENTIAL BENEFITS**

If you participate in this study, you may not expect any immediate benefits. Your participation will contribute to the medical knowledge regarding the use of wearables and the involvement of health coaches in shared decision-making in the management of chronic conditions.

ALTERNATIVE PROCEDURES/ TREATMENTS IF YOU DO NOT PARTICIPATE IN THE STUDY

There is no alternative procedure or treatment to the study procedures. You can choose not to take part in this study. The study procedures will not be carried out.

**COSTS & PAYMENTS IF PARTICIPATING IN THIS STUDY**

There should not be any additional cost incurred for participating in this research study.

The cost of your usual medical care (procedures, medications and doctor visits) will continue to be billed to you.

You will be reimbursed for your time and inconvenience as follows: $5 per visit for data collection after baseline (i.e., at the 3-month mark, at the 6-month mark, and at the 9-month mark of the study). The data collection point will be held on the day of your consultation to minimise inconvenience as far as possible.

If you take part in this study and you are randomized into the Intervention arm, you will be able to earn points via the EMPOWER mobile app by reaching certain achievements and milestones and use these points to redeem shopping and/or grocery vouchers of up to $120 in cumulative amount in the first 9 months of the study. There will be no voucher redemption after 9 months.

**INCIDENTAL FINDINGS**

There will not be any incidental findings arising in this research. “Incidental findings” are findings that have potential health or reproductive importance to research participants like you and are discovered in the course of conducting the study, but are unrelated to the purposes, objectives or variables of the study.

**PARTICIPANT’S RIGHTS**

Your participation in this study is entirely voluntary. You have a right to ask questions, which the study team will do their best to answer clearly and to your satisfaction.

In the event of any new information becomes available that may be relevant to your willingness to continue in this study, you (or your legal representative, if relevant) will be informed in a timely manner by the Principal Investigator or his/her representative and will be contacted for further consent if required.

**WITHDRAWAL FROM STUDY**

You are free to withdraw your consent and discontinue your participation in the study at any time, without your medical care being affected. If you decide to stop taking part in this study, you should tell the Clinical Research Coordinator.

If you withdraw from the study, or the intervention treatment is stopped for any reason,

- There will be no consequences.
- Please inform the Clinical Research Coordinator so that your data from the Smartphone wearable tracker will no longer be collected.
- The Smartphone wearable tracker smartwatch will have to be returned to the Clinical Research Coordinator.

However, any of your data that has been collected until the time of your withdrawal will be kept and analysed. The reason is to enable a complete and comprehensive evaluation of the study.

Your study doctor, the Principal Investigator of this study may stop your participation in the study at any time for one or more of the following reasons:

- Failure to follow the instructions of the Principal Investigator and/or study staff.
- The Principal Investigator decides that continuing your participation could be harmful to your health or safety.
- You require additional treatment that is not allowed in the study.
- The study is cancelled.

**RESEARCH RELATED INJURY AND COMPENSATION**

If you follow the directions of the Principal Investigator of this research study and you are injured due to the study research procedure given under the plan for the research study, our institution will provide you with the appropriate medical treatment.

Payment for management of the normally expected consequences of your treatment (i.e. consequences of your treatment that are not caused by your participation in the research study) will not be provided.

You still have all your legal rights. Nothing said here about treatment or compensation in any way alters your right to recover damages where you can prove negligence.

**CONFIDENTIALITY OF STUDY AND MEDICAL RECORDS**

Your participation in this study will involve the collection of Personal Data. “Personal Data” means data about you that makes you identifiable (i) from such data or (ii) from that data and other information which an organisation has or is likely to have access to. Examples of personal data include name, national registration identity card (NRIC), nationality, passport information, date of birth, and telephone number.

Personal Data collected for this study will be kept confidential. Your study records and medical records, to the extent required by the applicable laws and regulations, will not be made publicly available. Only the study team will have access to the personal data collected from you. In the event of any publication regarding this study, your identity will remain confidential.

However, the monitor(s), the auditor(s), the Institutional Review Board, and the regulatory authority(ies) will be granted direct access to your original medical records and study records to verify study procedures and data, without making any of your information public.

By signing the Consent Form, you consent to (i) the collection, access to, use and storage of your Personal Data by the study team, and (ii) the disclosure of such Personal Data to our authorised service providers and relevant third parties as mentioned above.

Any information containing your Personal Data that is collected for the purposes of this research will be stored in Singapore. To protect your identity, your Personal Data will be labelled with a unique code number. The code will be used in place of your name and other information that directly and easily identifies you. The study team will keep a separate file that links your code number to your Personal Data. This will be kept in a safe place with restricted access.

All data collected in this study are the property of the SingHealth Office of Regional Health. The data will be used for the purpose of this research study only unless you give permission for your data to be made available for future use in other research studies. In the event of any publication regarding this study, your identity will remain confidential.

By participating in this research study, you are confirming that you have read, understood and consent to the SingHealth Data Protection Policy, the full version of which is available at [www.singhealth.com.sg/pdpa](http://www.singhealth.com.sg/pdpa).

**WHO HAS REVIEWED THE STUDY**

This study has been reviewed by the SingHealth Centralised Institutional Review Board for ethics approval.

If you have questions about your rights as a participant, you can call the SingHealth Centralised Institutional Review Board at 8126 3660 during office hours (8:30 am to 5:30pm).

**WHO TO CONTACT IF YOU HAVE QUESTIONS REGARDING THE STUDY**

If you have questions about this research study or in the case of any injuries during the course of this study, you may contact:

**Principal Investigator**

**A/Prof Low Lian Leng**

**Department of Family Medicine & Continuing Care**

**6326 5872**

**SingHealth Polyclinics Hotline: 6350 7600**

If you have any feedback about this research study, you may contact the Principal Investigator or the SingHealth Centralised Institutional Review Board.

**CONSENT FORM FOR RESEARCH STUDY**

**Protocol Title:**

EMPOWERing patients with chronic diseases through smartphone app, health coaching and shared decision-making

**Principal Investigator:**

A/Prof Low Lian Leng

Department of Family Medicine & Continuing Care

Singapore General Hospital

I agree to participate in the research study as described and on the terms set out in the Participant Information Sheet.

The nature, risks and benefits of the study have been explained clearly to me and I fully understand them.

I understand the purpose and procedures of this study. I have been given the Participant Information Sheet and the opportunity to discuss and ask questions about this study and am satisfied with the information provided to me.

I understand that my participation is voluntary and that I am free to withdraw at any time, without giving any reasons and without my medical care being affected.

By participating in this research study, I confirm that I have read, understood and consent to the SingHealth Data Protection Policy.

____________________ ______________________________ ____________

Name of participant Signature/Thumbprint (Right / Left) Date of signing

**To be completed by parent / legal guardian / legal representative, where applicable**

I hereby give consent for _________________________ (Name of Participant) to participate in the research study. The nature, risks and benefits of the study have been explained clearly to me and I fully understand them.

I confirm that I have read, understood and consent to the SingHealth Data Protection Policy.

____________________ ____________________________ ______________

Name of participant’s Signature/Thumbprint (Right / Left) Date of signing

parent/ legal guardian/

legal representative

**To be completed by translator, if required**

The study has been explained to the participant/ legal representative in

________________________________by _____________________________________.

Language Name of translator

**To be completed by witness, where applicable**

I, the undersigned, certify that:

- I am 21 years of age or older.
- To the best of my knowledge, the participant or the participant’s legal representative signing this informed consent form had the study fully explained to him/her in a language understood by him/ her and clearly understands the nature, risks and benefits of the participant’s participation in the study.
- I have taken reasonable steps to ascertain the identity of the participant or the participant’s legal representative giving the consent.
- I have taken reasonable steps to ascertain that the consent has been given voluntarily without any coercion or intimidation.

Witnessed by: ________________________________ ___________________

Name of witness Date of signing

________________________________

Signature of witness

1. An impartial witness (who is 21 years of age or older, has mental capacity, who is independent of the research study, and cannot be unfairly influenced by people involved with the research study) should be present during the entire informed consent discussion if a participant or the participant’s legal representative is unable to read, and/or sign and date on the consent form (i.e. using the participant’s or legal representative’s thumbprint).After the written consent form and any written information to be provided to participant is read and explained to the participant or the participant’s legal representative, and after the participant or the participant’s legal representative has orally consented to the participant’s participation in the study and, if capable of doing so, has signed and personally dated the consent form, the witness should sign and personally date the consent form. This is applicable for Clinical Trials regulated by HSA and Human Biomedical Research under the HBRA.

2. For HBRA studies, the witness may be a member of the team carrying out the research only if a participant or the participant’s legal representative is able to read, sign and date on the consent form.

**Investigator’s Statement**

I, the undersigned, certify to the best of my knowledge that the participant/ participant’s legal representative signing this consent form had the study fully explained to him/her and clearly understands the nature, risks and benefits of the participant’s participation in the study.

_____________________ __________________________ ________________

Name of Investigator/ Signature Date

Person obtaining consent

**INFORMATION & CONSENT FORM FOR FUTURE RESEARCH**

This is an optional component that is separate from the research study. You may still participate in the research study if you say “No” to this. Please ask questions if you do not understand why we are asking for your permission.

*In this Consent Form for Future Research, we seek your permission to keep your data for future research. The data will be kept in SingHealth Office of Regional Health. Except if you withdraw your consent or there are limits imposed by law, there is no limit on the length of time we will store your data. Researchers will use your data for research long into the future.*

This is what will be done with your stored data:

- We may use the data to answer additional research questions in other research studies related to the use of mobile health (mHealth) technology, health coaching, shared decision making, hypertension management and diabetes management. This is outside the scope of the research study but is still related to diabetes and hypertension.
- We may share the data with other researchers at SingHealth institutions (Singapore General Hospital, SingHealth Polyclinics, etc.) but not with researchers outside of Singapore.
- *The stored data will be labelled with a code instead of information that directly identifies you (e.g. your name, NRIC, date of birth, etc.). We will keep a separate file (key) that links your code to your identifiable information.*
- *When we share your data with other researchers, it will be in a coded manner. They will not be able to identify you from the coded data.*
- *If you decide at a later time that you do not want your data to be used for future research, you can contact the Principal Investigator or study team at any time. All your stored data that has not been used or shared with other researchers will be removed and discontinued from further use, unless this information is already included in analyses or used in publications.*

*The use of your data in future research may result in intellectual property rights and commercial profits. If this should occur, you will not be compensated and will not receive any financial benefits or proprietary interest.*

**CONSENT FORM FOR FUTURE RESEARCH**

This component is optional. You do not have to agree to it in order to participate in the research study.

Please indicate your choice using the relevant checkbox.

- I do not agree to have my data stored for future use in other research studies.
- I agree to have my data stored for future use in other research studies.

I understand the purpose and nature of this optional component (storage of data for future use in other research studies). I have been given the Information & Consent Form for Future Research and the opportunity to discuss and ask questions about this optional component and am satisfied with the information provided to me.

I confirm that I have read, understood and consent to the SingHealth Data Protection Policy.

____________________ ___________________________ _____________

Name of participant Signature/Thumbprint (Right / Left) Date of signing

____________________ ____________________________ ______________

Name of participant’s Signature/Thumbprint (Right / Left) Date of signing

parent/ legal guardian/

legal representative

**To be completed by translator, if required**

The optional component (storage of data for future use in other research studies) has been explained to the participant/ participant’s legal representative in

________________________________by ___________________________________.

Language Name of translator

**To be completed by witness, where applicable**

I, the undersigned, certify that:

- I am 21 years of age or older.
- To the best of my knowledge, the participant or the participant’s legal representative signing this Information & Consent Form for Future Research had the optional component fully explained to him/her in a language understood by him/ her and clearly understands the purpose and the nature of this optional component.
- I have taken reasonable steps to ascertain the identity of the participant or the participant’s legal representative signing this Information & Consent Form for Future Research.
- I have taken reasonable steps to ascertain that the participant or the participant’s legal representative has not been coerced into giving consent.

Witnessed by: ________________________________ ___________________

Name of witness Date of signing

________________________________

Signature of witness

1. An impartial witness (who is 21 years of age or older, has mental capacity, who is independent of the research study, and cannot be unfairly influenced by people involved with the research study) should be present during the entire informed consent discussion if a participant or the participant’s legal representative is unable to read, and/or sign and date on the consent form (i.e. using the participant’s or legal representative’s thumbprint). After the written consent form and any written information to be provided to participant, is read and explained to the participant or the participant’s legal representative, and after the participant or the participant’s legal representative has orally consented to the participant’s participation in the study and, if capable of doing so, has signed and personally dated the consent form, the witness should sign and personally date the consent form. This is applicable for Clinical Trials regulated by HSA and Human Biomedical Research under the HBRA.

2. For HBRA studies, the witness may be a member of the team carrying out the research only if a participant or the participant’s legal representative is able to read, sign and date on the consent form.

**Investigator’s Statement**

I, the undersigned, certify to the best of my knowledge that the participant/ participant’s legal representative signing this Information & Consent Form for Future Research had the optional component (storage of data for future use in other research studies) fully explained to him/her and clearly understands the purpose and the nature of this optional component.

________________________ _______________________ ________________

Name of Investigator/ Signature Date

Person obtaining consent

**CONSENT TO BE CONTACTED FOR FUTURE RESEARCH**

*In this section, we seek your permission to contact you in the future for participation in research studies that you may be suitable for. Your information and contact details will be entered into a secured database at the SingHealth Office of Regional Health, and only personnel authorised by the Principal Investigator will have access to the database. If there is a particular research study that you may be suitable for, the Principal Investigator or study team members of the study will contact you to inform you about the research study.*

This component is optional. Your decision to be contacted for future research studies is completely voluntary and separate from your decision to participate in this study. Your decision will not affect your medical care or any benefits to which you are entitled. You may change your mind any time by contacting Ms Venice Liu at 6377 8465.

Please indicate your choice using the relevant checkbox.

- I do not agree to be contacted for future research.
- I agree to be contacted for future research that I may be eligible for. I agree to be contacted via _______________________________ (Phone/Email).

I understand the purpose and nature of this optional component (to be contacted for future research). I have been given the opportunity to discuss and ask questions about this optional component and am satisfied with the information provided to me.

I confirm that I have read, understood and consent to the SingHealth Data Protection Policy.

____________________ ___________________________ _____________

Name of participant Signature/Thumbprint (Right / Left) Date of signing

____________________ __________________________________________

Name of participant’s Signature/Thumbprint (Right / Left) Date of signing

parent/ legal guardian/

legal representative

**To be completed by translator, if required**

The optional component (to be contacted for future research) has been explained to the participant/ participant’s legal representative in

________________________________by ___________________________________.

Language Name of translator

**To be completed by witness, where applicable**

I, the undersigned, certify that:

- I am 21 years of age or older.
- To the best of my knowledge, the participant or the participant’s legal representative signing this Information & Consent Form for Future Research had the optional component fully explained to him/her in a language understood by him/ her and clearly understands the purpose and the nature of this optional component.
- I have taken reasonable steps to ascertain the identity of the participant or the participant’s legal representative signing this Information & Consent Form for Future Research.
- I have taken reasonable steps to ascertain that the participant or the participant’s legal representative has not been coerced into giving consent.

Witnessed by: ________________________________ ___________________

Name of witness Date of signing

________________________________

Signature of witness

1. An impartial witness (who is 21 years of age or older, has mental capacity, who is independent of the research study, and cannot be unfairly influenced by people involved with the research study) should be present during the entire informed consent discussion if a participant or the participant’s legal representative is unable to read, and/or sign and date on the consent form (i.e. using the participant’s or legal representative’s thumbprint). After the written consent form and any written information to be provided to participant, is read and explained to the participant or the participant’s legal representative, and after the participant or the participant’s legal representative has orally consented to the participant’s participation in the study and, if capable of doing so, has signed and personally dated the consent form, the witness should sign and personally date the consent form. This is applicable for Clinical Trials regulated by HSA and Human Biomedical Research under the HBRA.

2. For HBRA studies, the witness may be a member of the team carrying out the research only if a participant or the participant’s legal representative is able to read, sign and date on the consent form.

**Investigator’s Statement**

I, the undersigned, certify to the best of my knowledge that the participant/ participant’s legal representative signing this Information & Consent Form for Future Research had the optional component (to be contacted for future research) fully explained to him/her and clearly understands the purpose and the nature of this optional component.

________________________ _______________________ ________________

Name of Investigator/ Signature Date

Person obtaining consent
