# Supplementary material for: Empowering patients with comorbid diabetes and hypertension through a multi-component intervention of mobile app, health coaching and shared decision-making: Protocol for an effectiveness-implementation of randomised controlled trial
Source: PLoS One. 2024 Feb 26;19(2):e0296338. doi: 10.1371/journal.pone.0296338 (PMC10896544; doi:10.1371/journal.pone.0296338)
Supplement: S3 File — (DOCX) [file pone.0296338.s003.docx]

Supplementary information 3: EMPOWER app features

| App features | Description | Intervention function* |
| --- | --- | --- |
| Nudges | Personalised notifications generated based on the user’s inputs | Enablement, Education, Persuasion |
| Gamification | Monetary reward for logging, wearing smartwatch and achieving health goal | Incentivisation |
| Educational resources | Instant access to diabetes and hypertension management information | Education, Training |
| Log and report | Monitor physical activity, diet, medication, weight, blood glucose and blood pressure readings | Education |

* Based on the behavioural change wheel
