# Supplementary material for: Empowering patients with comorbid diabetes and hypertension through a multi-component intervention of mobile app, health coaching and shared decision-making: Protocol for an effectiveness-implementation of randomised controlled trial
Source: PLoS One. 2024 Feb 26;19(2):e0296338. doi: 10.1371/journal.pone.0296338 (PMC10896544; doi:10.1371/journal.pone.0296338)
Supplement: S4 File — (DOCX) [file pone.0296338.s004.docx]

Supplementary information 4: Sample interview questions for patients

| Sample interview questions | TDF Domain(s) | SCT Domain(s) |
| --- | --- | --- |
| 1. Can you share with me what you know about health coaching? 2. How was your interaction with the health coach on your chronic disease management? Can you tell me more? (Prompt: knowledge on condition, skills in managing chronic condition, confidence in managing chronic condition, beliefs on outcomes) 3. Can you share anything that facilitated your interaction with health coach? 4. Can you share with me what do you know about EMPOWER mobile app? 5. What are your thoughts on the EMPOWER mobile app? (Prompt: reward, knowledge on condition, skills in managing chronic condition, confidence in managing chronic condition, beliefs on outcomes) 6. What types of training was (and would be) useful for users of EMPOWER app? 7. Can you share other ways to improve chronic disease management? | Knowledge & Skills & Beliefs about capabilities & Beliefs about consequences  & Reinforcement | Knowledge & Skill & Self-efficacy  & Outcome expectation  & Reinforcement |
| 1. What is your goal for your chronic disease management? 2. What are your thoughts on your goal setting with the health coach? 3. What are your thoughts on your goal setting with your doctor? 4. What are your thoughts on the goal setting feature in the EMPOWER app? | Goals | Intention |
| 1. How did health coaching alter (or did not alter) how you manage your chronic disease? (Prompt: stages of change) 2. How did community nursing alter (or did not alter) how you manage your chronic disease? (Prompt: stages of change) 3. How did EMPOWER app alter (or did not alter) how you manage your chronic disease? (Prompt: stages of change) | Intentions | Intention |
| 1. What things influenced whether or not you interacted with coach? (eg, cost, competing tasks or time constraints, social support)   How can we help address these issues?   1. What things influenced whether or not you interacted with nurse? (eg, cost, competing tasks or time constraints, social support)   How can we help address these issues?   1. What things influenced whether or not you used the EMPOWER app? (eg, Wi-Fi availability, equipment to complete, competing tasks or time constraints, social support)   If yes, how can we help address these issues? | Environmental context and resources & Social influences | Barriers and opportunities & Social support |

Abbreviation

SCT: Social cognitive theory; TDF: theoretical domain framework
