# Supplementary material for: Empowering patients with comorbid diabetes and hypertension through a multi-component intervention of mobile app, health coaching and shared decision-making: Protocol for an effectiveness-implementation of randomised controlled trial
Source: PLoS One. 2024 Feb 26;19(2):e0296338. doi: 10.1371/journal.pone.0296338 (PMC10896544; doi:10.1371/journal.pone.0296338)
Supplement: S5 File — (DOCX) [file pone.0296338.s005.docx]

Supplementary information 5: Sample interview questions for other stakeholders

| Sample interview questions | CFIR Domain(s) |
| --- | --- |
| 1. What are your thoughts on this program? (Prompt: what do you think were the advantages or drawbacks compared to current practice? Complexity? Design?)   *to get feedback on health coaching, EMPOWER app  Any adaptations done to this program so far? | Innovation  Construct(s): Innovation relative advantage, Innovation adaptability, Innovation complexity, Innovation design |
| 1. Can you tell us what you know about any other organizations that have implemented or are intending to implement similar interventions? | Outer setting  Construct(s): External Pressure |
| 1. How well did the implementation of this program fit with existing work processes and practices in your organization? 2. Can you describe how this program could be further integrated into current processes in healthcare? 3. Can this program replace or complement a current program or process? 4. What are your thoughts on the content in this program? (Prompt: important to the recipients?) 5. Any gaps in the existing work processes and practices in community for chronic disease management? How can the health coaching address these gaps? | Inner setting  Construct(s): Tension for change, Compatibility |
| 1. Do you have sufficient support from other stakeholders? (e.g., administrators, patients) | Individuals  Construct(s): Other Implementation support, Innovation recipients |
| 1. Did you expect to have sufficient resources to administer coaching intervention? If so, what resources were you counting on? If not, what resources would you need? (prompt: competence, knowledge, skills) 2. Did you expect to have sufficient opportunity to implement and administer coaching intervention? (prompt: availability, scope and power) 3. What was your motivation to join this role? | Characteristics  Construct(s): Capability, opportunity, motivation |
| 1. Any way that we can encourage other healthcare professionals to participate in this coaching role? 2. Any way that we can encourage patients to participate in health coaching? 3. Any way that we can encourage patients to use EMPOWER app? 4. Can you share your thoughts on strategies to improve the implementation, scaling up and sustainability of this program? | Implementation process  Construct(s): Engaging |

Abbreviation

CFIR: Consolidated Framework for Implementation Research
